# Autophagy-mediated turnover of Dynamin-related Protein 1

**DOI:** 10.1186/1471-2202-14-86

**Published:** 2013-08-09

**Authors:** Phillip R Purnell, Howard S Fox

**Affiliations:** 1 Department of Pharmacology and Experimental Neuroscience, University of Nebraska Medical Center, Omaha, NE, USA

**Keywords:** Drp1, Autophagy, Neuron

## Abstract

**Background:**

Drp1 is the primary protein responsible for mitochondrial fission. Perturbations of mitochondrial morphology and increased fission are seen in neurodegeneration. While Drp1 degradation induced by Parkin overexpression can be prevented by proteasome inhibition, there are numerous links between proteasomal and autophagic processes in mitochondrial protein degradation. Here we investigated the role of autophagy in Drp1 regulation.

**Results:**

We demonstrate that autophagy plays a major role in the control of Drp1 levels. In HEK-293T cells, inhibitors of autophagy increase total Drp1 and levels of Drp1 in the mitochondrial cellular fraction. Similarly by silencing *ATG7*, which is required for initiation of autophagy, there is an increased level of Drp1. Because of the role of increased Drp1 in neurodegeneration, we then examined the ability to modulate Drp1 levels in neurons by inducing autophagy. We are able to decrease Drp1 levels in a time- and dose-dependent manner with the potent neuronal autophagy inducer 10-NCP, as well as structurally related compounds. Further, 10-NCP was able increase average mitochondrial size and length verifying a functional result of Drp1 depletion in these neurons.

**Conclusions:**

These pharmacological and genetic approaches indicate that autophagy targets Drp1 for lysosomal degradation. Additionally these data suggest a mechanism, through Drp1 downregulation, which may partly explain the ability of autophagy to have a neuroprotective effect.

## Background

Mitochondria are dynamic organelles undergoing constant changes in size and shape. This perpetual cycle of fusion and fission is a delicate balancing act and is critical for proper cellular function [1]. Mitochondrial morphology is regulated by the opposing actions of fusion and fission proteins. The central regulators of fusion are the mitofusions (Mfn1 and Mfn2). These proteins are necessary to prevent unchecked fission, which can lead to hyper fragmented mitochondria [2]. The principal protein responsible for fission is Drp1 (Dynamin-related protein 1). Several groups have specifically linked Drp1 and excessive mitochondrial fragmentation to neurodegeneration [3]. First discovered and characterized in yeast (*DNM1P*) [4], human Drp1 (*DNM1L*) was shown to promote mitochondrial fission in human cells [5]. Drp1 is a primarily cytoplasmic protein which, when activated, can form ring-like multimers and translocate to the mitochondria. There, in concert with accessory proteins, Drp1 facilitates mitochondrial scission [6]. Drp1 activity and cellular localization are highly regulated by post-translational modifications including phosphorylation, ubiquitination, sumoylation, and N-linked nitrosylation [7]. Here we characterize the ability of autophagy to regulate protein expression of Drp1.

Macroautophagy, here referred to as autophagy, is a mechanism of degradation and recycling of proteins beginning with sequestration of cytoplasmic proteins into double-membrane vesicles followed by fusion with the lysosome and subsequent protein degradation. Autophagy is initiated by multiple cellular cues including starvation and has extensive roles in numerous cellular processes and pathophysiologic mechanisms [8]. First characterized in yeast, autophagy is regulated by a highly conserved family of *ATG* (autophagy-related) genes. In mammalian cells, the process can be actuated by mTOR inhibition as well as mTOR-independent mechanisms. Interestingly, elongation of the autophagic membrane involves two ubiquitin-like conjugation systems where Atg7 (an E1-like enzyme) works in conjunction with Atg3 or Atg10 (both E2-like) to conjugate Atg5 to Atg12 and light chain 3 (LC3, homolog of Atg8) to phosphatidylethanolamine (PE), respectively. Atg7-deficient cells display impaired autophagosome formation and lysosomal protein turnover [9]. While the non-PE conjugated form of LC3 (known as LC3-I) is present in the cytoplasm, the PE-conjugated form (LC3-II) is then attached to the autophagosomes and represents a marker for autophagosomes. The autophagosomes then deliver their contents to the lysosome via fusion. Experimentally, several chemical inhibitors, including bafilomycin A1 and chloroquine, can inhibit this autophagosomal-lysosomal fusion. Autophagy specific for mitochondria, termed mitophagy, has also been shown to play an increasingly important role in regulation of mitochondrial dynamics [10].

Various lines of evidence point to altered mitochondrial dynamics as an underlying pathologic mechanism contributing to many neurodegenerative conditions including Alzheimer’s (AD), Parkinson’s (PD), and Huntington’s (HD) diseases [11,12]. Because of their metabolic requirements, neurons are exquisitely dependent on proper mitochondrial function including appropriate fusion and fission. Mutations of the principal proteins responsible for mitochondrial fusion and fission are seen in several neurological disorders. *MFN2* mutations are seen in patients with Charcot-Marie Tooth Neuropathy Type 2 [13]. Opa1, a protein responsible for inner mitochondrial membrane fusion, is mutated in autosomal dominant Optic Atrophy Type 1 [14,15]. A neonatal lethal mutation in *DNM1L*, which inhibits proper Drp1 mitochondrial assembly, was characterized in a patient with microcephaly, abnormal brain development, and optic atrophy [16,17]. Several groups have highlighted the importance of Drp1 function in neurodegeneration [3]. In a mouse model of AD, neuronal Drp1 is upregulated and increased Drp1 activity is thought to occur in the brains of AD patients, which may contribute to the characteristic pathology [18,19]. In HD, Drp1 associates with mutant Huntington protein altering normal mitochondrial axonal movement [20]. In a PD neuroblastoma cell line model, downregulation of Drp1 protects from the neurotoxic effects of 6-hydroxydopamine [21]. Work by several groups investigating familial PD, often caused by *PARK2* or *PINK1* mutations, shows autophagy serves a neuroprotective role and that autophagy is in part responsible for mitochondrial morphology, additionally these groups have found increased fission these PD model systems [22-24]. Together these data support a role of increased Drp1 and increased Drp1 activity as a culprit in neurodegeneration. Here we characterize a general mechanism of Drp1 turnover through autophagy. We show that autophagic regulation may be exploited using existing FDA-approved compounds to lower Drp1 levels in neurons.

## Results and discussion

### Inhibition of autophagosomal-lysosomal degradation increases Drp1 Levels

**Figure 1:**
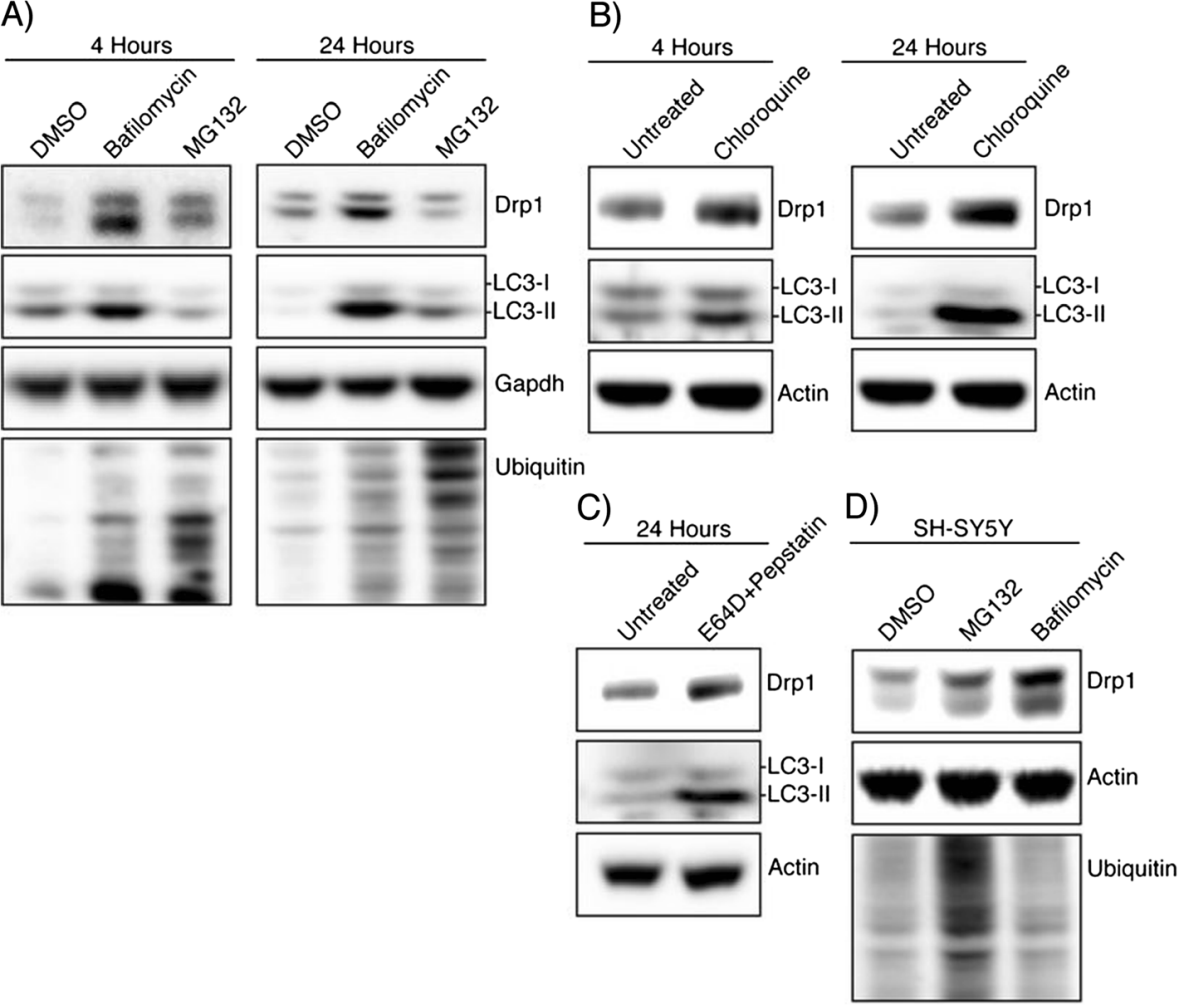
**Drp1 expression is increased after inhibiting autophagy. (A)** HEK-293T cells were incubated with DMSO, Bafilomycin (100 nM), or MG132 (25 μM) for 4 hours and 24 hours and whole cell lysate was analyzed by Western blotting using the indicated antibodies. LC3 levels confirm the bafilomycin is inhibiting autophagic turnover and total ubiquitin levels confirm MG132 effect. **(B)** HEK-293T cells were incubated with chloroquine (10 μM) for 4 hours and 24 hours followed by Western blotting **(C)** Lysosomal protease inhibitors E64D (10 μg/ml) and pepstatin (10 μg/ml) were incubated with HEK-293T cells for 24 hours. **(D)** SH-SY5Y cells were incubated with DMSO, bafilomycin (100 nM), or MG132 (25 μM) for 24 hours.

To understand how autophagy affects the endogenous levels of Drp1, we used bafilomycin A1 to decrease autophagic protein degradation. Bafilomycin inhibits the lysosomal vacuolar H+ ATPase and autophagosomal-lysosomal fusion and is a commonly used inhibitor of autophagic degradation. Bafilomycin treatment in HEK-293T cells increased the expression of Drp1 at both 4 and 24 hours (Figure 1A). To ensure the efficacy of this bafilomycin treatment to inhibit autophagic turnover, levels of LC3 were also analyzed, confirming inhibition of autophagosomal-lysosomal fusion with increased LC3-II levels. To investigate whether proteasomal inhibition would have a similar effect, as previously reported [25], we analyzed the effect of the proteasome-specific inhibitor MG132. Treatment with MG132 did increase the levels of Drp1 at 4 hours, although not as dramatically as bafilomycin treatment. These data suggested that although proteasomal-mediated turnover of Drp1 occurs in these cells, inhibiting autophagic turnover has a more dramatic effect on Drp1 protein expression levels. To confirm that this is not a bafilomycin-specific effect, treatments with other lysosomal inhibitors, chloroquine (autophagosomal-lysosomal fusion inhibitor) and a combination of the lysosomal protease inhibitors, pepstatin and E64D, were used. At both 4 and 24 hours there were increased levels of Drp1 in chloroquine-treated cells corresponding with increased LC3-II levels (Figure 1B). Similarly, the combination of E64D and pepstatin was able to increase the level of Drp1 protein in HEK-293T cells after 24 hours (Figure 1C). To determine if this effect is cell line dependent, SH-SY5Y were treated with a similar regimen of bafilomycin and MG132 (Figure 1D). After 24 hours, a comparable increase in Drp1 level was seen after bafilomycin treatment, a lower level of increase was also seen in the MG132 treated cells. The data from the combination of autophagic inhibitors and multiple cell lines together suggest levels of Drp1 expression are considerably dependent on autophagy.

### Blocking autophagy increases the mitochondrial fraction of Drp1

**Figure 2:**
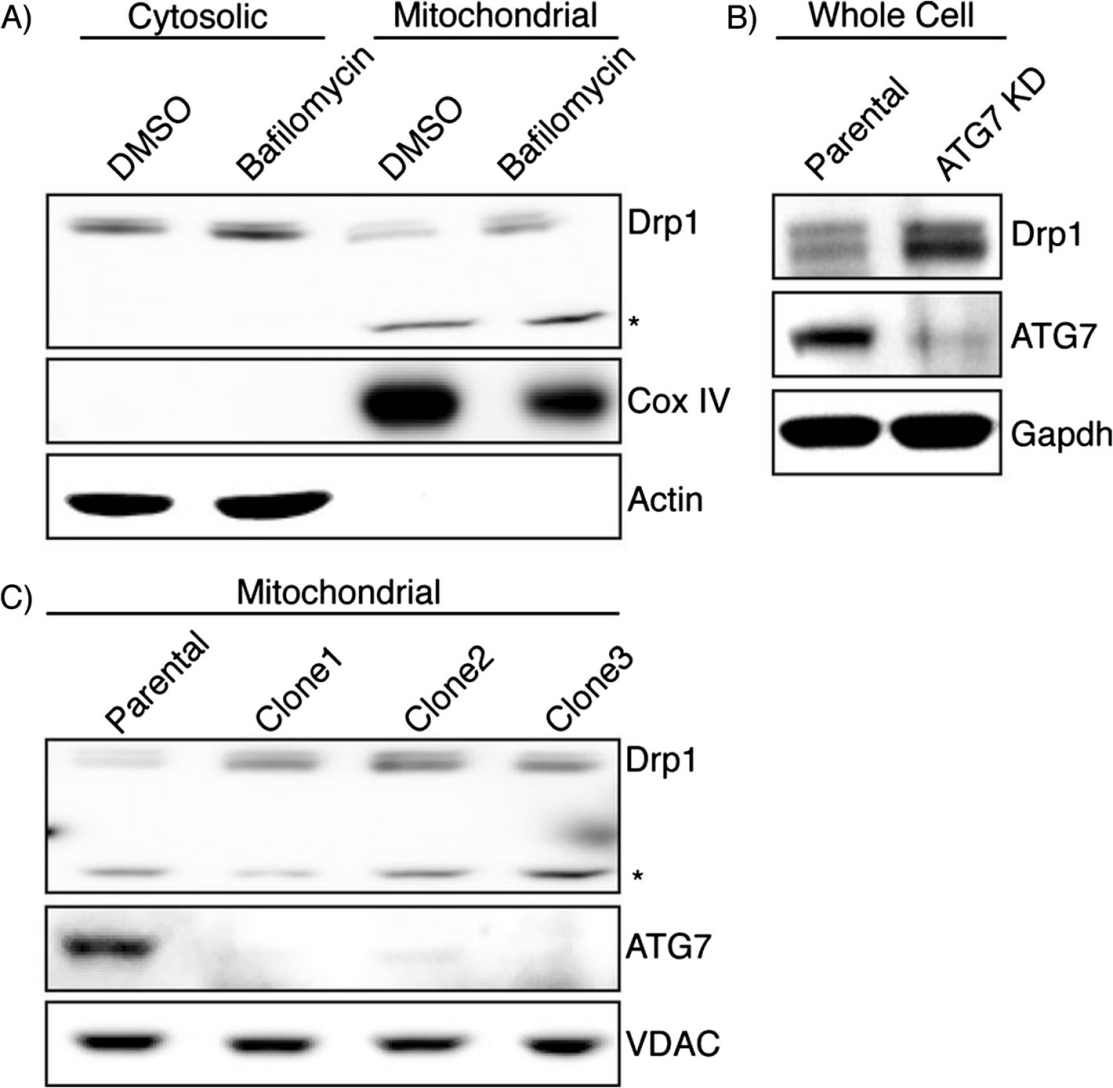
**Bafilomycin and *ATG7 *knockdown increases mitochondrial Drp1. (A)** Cytosolic and mitochondrial fractions from bafilomycin treated HEK-293T cells were isolated by differential centrifugation and analyzed by immunoblotting. *Drp1 antibody reactive band that is only seen in the mitochondrial fraction. Cox IV is used as a mitochondrial loading control and Actin indicates most of the cytosol is absent from the mitochondrial fraction. **(B)***ATG7* stable knockdown clones were created and whole cell lysate was subject to immunoblotting. **(C)** Mitochondrial fractions of *ATG7* knockdown clones were isolated and lysates were probed with the indicated antibodies. VDAC is used as a mitochondrial loading control.

In order to determine if blocking autophagy is also increasing the levels of the mitochondrially-associated Drp1, cytosolic and mitochondrial fractions of HEK-293T cells treated with bafilomycin were isolated. Bafilomycin increased Drp1 in both the cytosolic and mitochondrial fraction in these cells (Figure 2A). To ensure that the increase of Drp1 was specifically autophagy-mediated we created stable *ATG7* knockdown HEK-293T cells. HEK-293T cells were transfected with an *ATG7* shRNA plasmid and transfected cells were selected with hygromycin. These knockdown cells again demonstrated an increased Drp1 level after autophagy is perturbed by *ATG7* knockdown (Figure 2B). Next we selected several different *ATG7* knockdown clones and analyzed the levels of Drp1 in the mitochondrial fraction by immunoblotting (Figure 2C). Each of the clones demonstrated an increased Drp1 level in the mitochondrial fraction. Taken together, the results from both the pharmacological and genetic approaches to inhibiting autophagy suggest not only does autophagy regulate overall Drp1 levels but, autophagy is responsible for the levels of mitochondrially-associated Drp1, the cellular pool ultimately responsible for fission.

### Induction of neuronal autophagy lowers endogenous Drp1 levels

**Figure 3:**
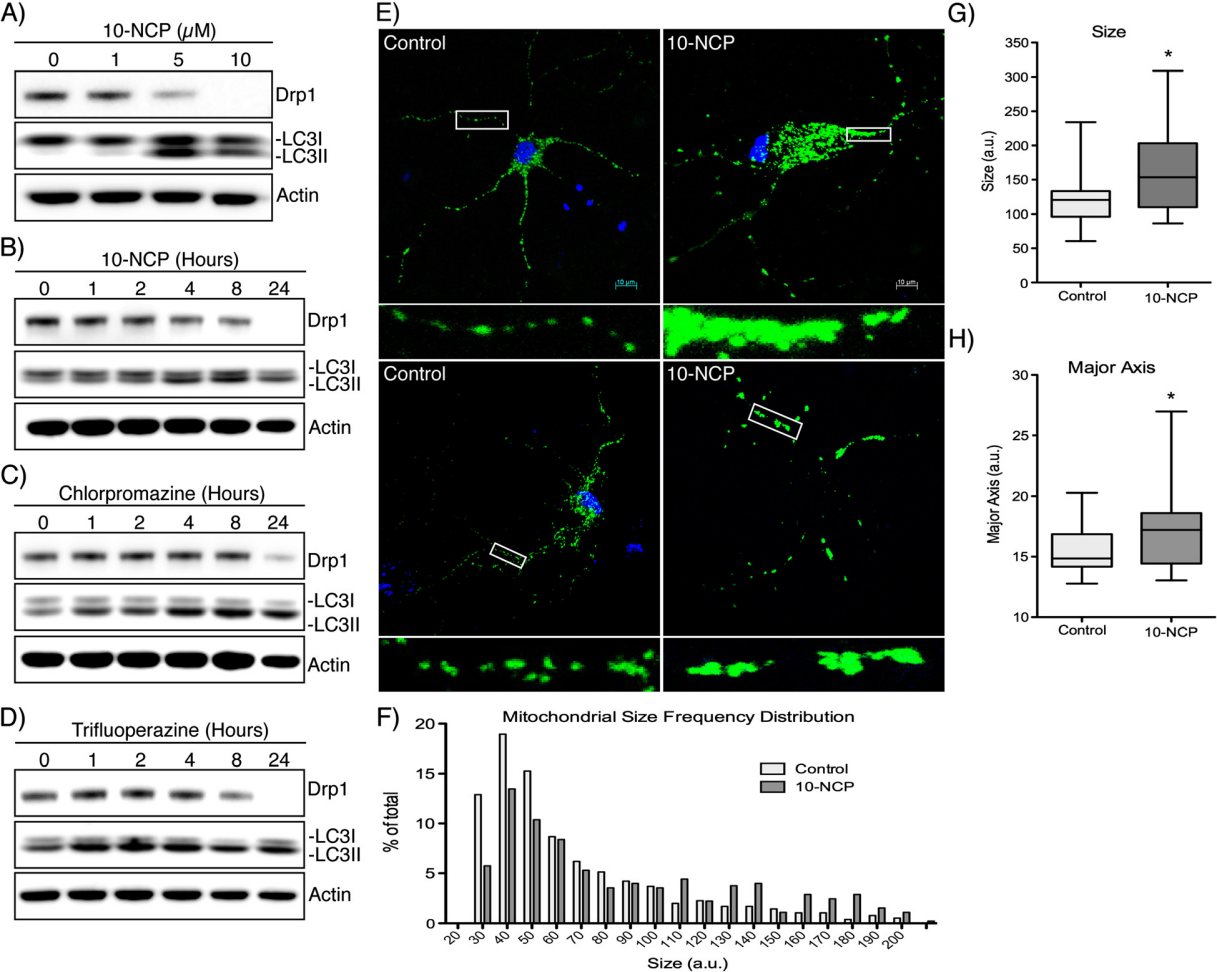
**Inducing autophagy decreases Drp1 levels in rat-derived striatal neurons. (A)** E18 rat-derived striatal neurons were incubated with 10-NCP at the concentrations indicated for 12 hours. **(B)** Neurons were incubated with 5 μM 10-NCP for the indicated times. **(C** and **D)** Chlorpromazine and Trifluoperazine (5 μM) was incubated for the times indicated followed by Western blotting. **(E)** Striatal neurons were incubated with 10-NCP for 12 hours along with transduction of CellLight Mitochondria GFP to allow labeling and visualization of mitochondria. **(F)** Frequency distribution of mitochondrial size in control and 10-NCP treated neurons as quantitated from confocal immunofluorescent images using the Mitochondrial Morphology plug-in for ImageJ (n > 500 for each condition, X-axis truncated at 200). **(G** and **H)** Box and whisker plot (with whiskers representing 5-95^th^ percentile) of the average mitochondrial size and major axis per neuron (n = 20). *P < 0.05.

Imbalanced mitochondrial fusion and fission has been well characterized in neurodegeneration [26]. Increased Drp1 levels in AD [18] and HD patients [27], and cellular data from PD models all suggest that increased Drp1 is associated with pathogenesis. With this and evidence for autophagy-mediated turnover of Drp1 in mind, we studied the possible effect of inducing autophagy in a primary neurons. The compound 10-NCP has been found to potently induce autophagy in primary neurons [28]. Using cultured E18 rat-derived striatal neurons, 10-NCP is able to reduce Drp1 levels in a dose-dependent manner with a corresponding increase in LC3-II levels (Figure 3A*)*. This effect is also time dependent and Drp1 is effectively decreased by 24 hours of 10-NCP treatment (Figure 3B). The previous study of 10-NCP in neurons also identified several FDA-approved structural analogs, which were able to analogously induce autophagy [28]. We were interested to see if these compounds would act similarly to 10-NCP in these neurons. Both compounds tested (chlorpromazine and trifluoperazine) were able to decrease Drp1 in a similar time dependent manner to 10-NCP (Figure 3C-D). To confirm the functional relevance of Drp1 downregulation, E18 rat-derived striatal neurons were treated with vehicle or 10-NCP along with transduction of a mitochondrially-targeted GFP (Figure 3E). It appears that 10-NCP leads to decreased fission as mitochondria become more interconnected after treatment (Figure 3E). To quantify this effect, mitochondrial morphology was analyzed using a well-characterized image analysis program (ImageJ plug-in) [22]. After 10-NCP treatments, mitochondrial size is increased in a large percentage of the neuronal mitochondria (Figure 3F). To better quantify this effect, the average and distribution of mitochondrial size and length (defined by the major mitochondrial axis) in control and 10-NCP treated neurons is shown (Figure 3G-H). These data suggest the induction of autophagy in neurons is able to decrease Drp1 and this approach may be useful to decrease the aberrant Drp1 expression observed in neurodegeneration.

## Conclusions

Drp1 is the primary protein responsible for mitochondrial fission and as many groups have shown, increased Drp1 and increased fission are well characterized in several neurodegenerative diseases [3,12]. In this study we have demonstrated a pathway for Drp1 autophagic degradation. Chemical inhibition of lysosomal degradation and *ATG7* knockdown increased Drp1 levels. Further, activation of autophagy in neurons with multiple compounds, including two that are already FDA approved, were able to decrease Drp1 levels. We demonstrate that Drp1 protein turnover is not limited to one proteolytic system and there is likely interplay between the autophagic system and the UPS in order to finely tune Drp1 expression. The ubiquitination of Drp1 by Parkin [25] and the ability of Parkin to induce both K48- and K63-linked ubiquitin chains [29], certainly hints to the importance of the interaction and balance between these two systems. In our neuronal system however, Drp1 levels are quite sensitive to induction of autophagy. We hypothesize that the neuroprotective effect of autophagy inducers may in part be due to their ability to reduce Drp1 mediated mitochondrial fission effectively counteracting the increased fission observed in neurodegeneration.

## Methods

### Chemicals and cell line culture

Bafilomycin A1 and MG132 were from Tocris Bioscience. Chloroquine, Chlorpromazine, E64D, Pepstain, and Trifluoperazine were from Sigma. 10-NCP was from EMD Millipore. The following drug concentrations were used unless noted: Bafilomycin (100 nM in DMSO), MG132 (25 μM in DMSO), Chloroquine (10 μM in H_2_O), Chlorpromazine (5 μM in H20), Trifluoperazine (5 μM in H_2_0), and 10-NCP (5 μM in H_2_0). HEK-293T cells were maintained in DMEM with 10% fetal bovine serum and SH-SY5Y were maintained in DMEM/F12 with 10% fetal bovine serum, both were obtained from the American Type Culture Collection (ATCC).

### Antibodies

The following antibodies and concentrations were used for Western blotting. Drp1 (5391, 1:2500, Cell Signaling Technology), LC3 (M152-3B, 1:5,000, MBL), Ubiquitin (3936, 1:2500, Cell Signaling Technology), Actin (A2668, 1:20,000, Sigma), GAPDH (437000, 1:20,000, Sigma), and ATG7 (sc-33211, 1:1000, Santa Cruz).

### Mitochondrial isolation and Western blotting

Whole cells were harvested by rinsing with cold PBS, scraped, and lysed with CelLytic M (Sigma). Mitochondria were isolated using a mitochondrial isolation kit for cultured cells using the standard protocol (Mitosciences). Briefly, 1×10^7^cells were homogenized and resuspended in lysis buffer, followed by low speed centrifugation. Cells were once again homogenized followed by a high-speed (12,000 x g) centrifugation to collect mitochondria. Supernatant before the mitochondria isolation step was collected as the cytosolic fraction. Equal amounts of protein were resolved by SDS-PAGE and transferred to nitrocellulose. Membranes were then blocked and incubated at 4°C overnight with primary antibodies. Membranes were then rinsed and incubated for 1–2 hours at RT with appropriate anti-mouse or anti-rabbit HRP-conjugated secondary antibodies (EMD Millipore) followed by incubation with SuperSignal Chemiluminescent Substrate (Pierce). Chemiluminescent images were acquired on an Image Station 4000MM (Carestream Molecular Imaging).

### Generation of *ATG7* knockdown cell lines

To generate stable *ATG7*-deficient cells, HEK-293T cells were transfected with *ATG7* shRNA encoding plasmids (pSilencer2.1-U6 Hygro vector, Ambion) targeted to the following sequences of human *ATG7*: ACAAGAGAAAGCTGGTCATCAA and AAGGAGTCACAGCTCTTCCTT (Kindly provided Dr. Hui Pan, UNMC). Stable clones were then selected with cloning discs followed by growth in 250 μg/ml hygromycin B containing media for 21 days. After selection, knockdown was verified by immunoblotting (Figure 2A).

### Isolation and culture of primary neurons

All animal experiments were performed following NIH guidelines under approval from the UNMC IACUC. E-18 rat striatal neurons were isolated using a previously established protocol and plated as described onto poly-D lysine coated coverslips [30]. Treatments with 10-NCP, chlorpromazine and trifluoperazine were performed after 14 days in culture.

### Mitochondrial imaging of primary neurons

Fourteen days after plating, cells were incubated with a half-exchange of media alone or media containing 10-NCP and CellLight Mitochondria GFP (Invitrogen) for 12 hours. Neurons were then fixed for 20 minutes with 3.7% paraformaldehyde and mounted with ProlongGold containing DAPI (Invitrogen). Slides were imaged on a Zeiss 710 Confocal Laser Scanning Microscope at 63× and Zeiss Zen imaging software. For quantification of mitochondrial morphology a workflow was used based on the methods described by the Strack group [31]. Briefly, the mitochondrial labeled channel was isolated and converted to a monochrome image. This image was then 2-D deconvolved using the Iterative Deconvolution ImageJ plug-in (Bob Dougherty, http://www.optinav.com/imagej.html Iterative), and converted to a binary image in ImageJ. Finally, the Mito-Morphology Macro was used to quantitate shape [22].

### Statistical analysis

Where appropriate, experiments were performed three independent times; values represent mean plus standard error of triplicate experiments. Two-sided P-values < 0.05 were considered significant.

## Competing interests

Both authors declare they have no competing interests.

## Authors’ contributions

PP carried out all assays and laboratory work. PP and HS were responsible for data interpretation. PP and HS were responsible for experimental design. PP and HS drafted the manuscript. PP and HS approved the final draft of this manuscript. All authors read and approved the final manuscript.
